# Anti-Glutamic Acid Decarboxylase (GAD) 65 Encephalitis Mistaken for Herpes Encephalitis and Hashimoto’s Encephalitis (HE): A Case Report

**DOI:** 10.7759/cureus.35365

**Published:** 2023-02-23

**Authors:** Mohammad Abu-Abaa, Ghassan Al-Qaysi, Ali Abdulsahib, Omar Jumaah, Andre Ruppel

**Affiliations:** 1 Internal Medicine, Capital Health Regional Medical Center, Trenton, USA

**Keywords:** status epilepticus, refractory seizure, herpes virus encephalitis, hashimoto’s encephalopathy, anti-gad 65 encephalitis

## Abstract

Autoimmune encephalitis is increasingly recognized in clinical practice. We are presenting a 72-year-old female patient who initially presented with a new onset seizure with temporal lobe abnormality on imaging. This was initially attributed to herpes encephalitis although herpes polymerase chain reaction (PCR) was negative. The patient was treated with acyclovir and antiepileptic medication (AEM) with some clinical improvement. She presented again with refractory seizure evolving to status epilepticus. Escalation of AEMs was pursued and positive anti-thyroid peroxidase (TPO) antibody prompted consideration of Hashimoto’s encephalitis (HE) and treatment with high-dose corticosteroids and intravenous immunoglobulin (IVIG). However, poor response to steroid argued against HE, and extended autoimmune encephalitis panel revealed positive anti-glutamic acid decarboxylase (GAD) antibody. This case raises the clinical pearl that anti-thyroid antibodies, e.g anti-TPO antibody, can be seen in those with autoimmune encephalopathies other than HE and HE remains a diagnosis of exclusion. It also helps to remind clinicians that a new onset refractory seizure even with temporal lobe changes is not pathognomonic for herpes encephalitis, and although negative serology does not rule out the diagnosis, it is recommended to rule out autoimmune encephalitis as it shares similar clinical and radiological picture.

## Introduction

Three antibodies have been reported in association with non-paraneoplastic autoimmune encephalitis including voltage gated potassium channels (VGKC), N-methyl-D-aspartate (NMDA) receptor and glutamic acid decarboxylase (GAD) antibodies. GAD enzyme is the rate limiting enzyme responsible for conversion of glutamate to gamma-aminobutyric acid (GABA) [1]. Anti-GAD antibody has two isotypes, the most common anti-GAD 65 and less common anti-GAD 67. Anti-GAD 65 antibody is a marker of type 1 diabetes [2]. Hashimoto’s encephalitis (HE) is another type of autoimmune encephalitis that is characterized by encephalopathy, seizure, positive antithyroid antibody, and good response to steroid therapy [3]. Although MRI findings in HE are variable, new onset seizure and temporal lobe imaging changes can be seen in HE, in anti-GAD 65 encephalitis, and also in herpes encephalitis.

## Case presentation

**Figure 1:**
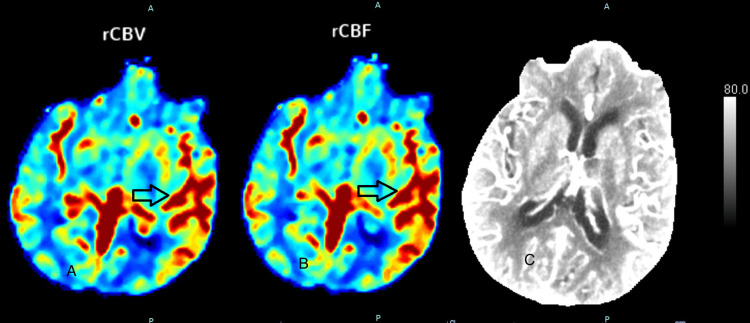
Brain CT perfusion scan (A) is  cerebral blood volume (CBV) and (B) is cerebral blood flow (CBF). Both CBV and CBF show increase in perfusion of the left temporal lobe without any corresponding vascular defect in (C).

**Figure 2:**
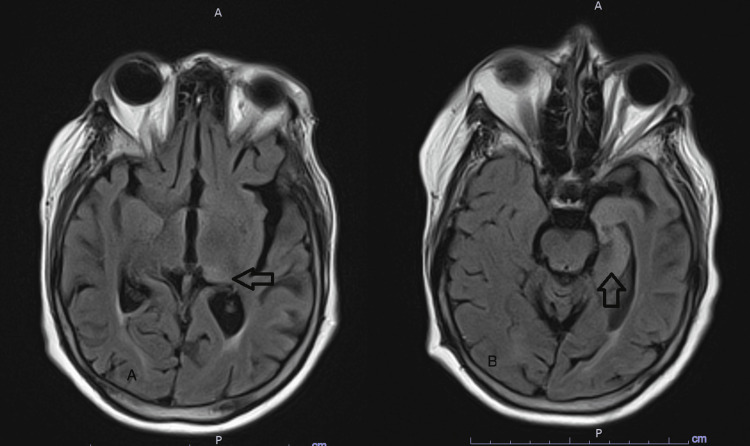
Initial MRI brain findings MRI brain showing areas of hyper-intensity on fluid attenuation inversion recovery (FLAIR) sequence in left thalamus (arrow in A) and left hippocampus (arrow in B).

A 72-year-old white female patient presented to the emergency department (ED) with right-sided weakness, slurred speech, and a new onset complex partial seizure affecting the right sided extremities that started around 15 hours prior to presentation. Past medical history is remarkable for atrial fibrillation on carvedilol and apixaban and depression on sertraline. No prior history of seizure, thyroid disorders, or diabetes was reported. Vitals signs included temperature of 36.9°C, blood pressure of 120/70 mmHg, heart rate of 165 beats per minute and oxygen saturation (SpO_2_) of 97% on room air. Physical examination showed lethargy, limited orientation to self, impaired recent memory, impaired concentration, conjugate gaze deviation to the left, muscle power 2/5 on right upper and 3/5 right lower extremities, 5/5 on left upper and lower extremities, right-sided hemineglect, evidence of tongue bite, and grossly intact cranial nerves with no facial droop. Electrocardiography (EKG) showed atrial fibrillation with rapid ventricular response that was rate controlled with beta blockers. CT head ruled out cranial bleeding. CT perfusion study brain showed increased perfusion of the left temporal lobe compatible with seizure history (Figure 1). MRI brain without contrast showed fluid attenuation inversion recovery (FLAIR) hyperintensity with equivocal restricted diffusion in the left temporal lobe involving the left hippocampus and thalamus (Figure 2). Basic lab work up showed only leukocytosis of 17,000 cells/ml and thrombocytosis of 822,000 cells/ml. Thyroid function test was within normal limits. Drug screen was negative. She was started on levetiracetam. Improvement of muscle power on the right side was evident within 24 hours of admission suggestive of postictal Todd’s paralysis.

Lumbar puncture (LP) with cerebrospinal fluid (CSF) analysis showed clear fluid with elevated white blood cell count (WBC) 8 cells/ml, protein 95 mg/dl, and glucose 71 mg/dl. Meningitis panel was nonreactive for Cryptococcus, Cytomegalovirus, Enterovirus, E. Coli, Haemophilus influenzae, Herpes Simplex virus (HSV) 1,2 and 6, Human parechovirus, Listeria, Neisseria, Streptococci and Varicella Zoster virus. West Nile Virus, Lyme serology and venereal disease research laboratory (VDRL) were also negative. CSF culture remained negative and the patient was started on acyclovir for presumed HSV encephalitis. Continuous electroencephalography (EEG) showed periodic lateralized epileptiform discharges (PLEDS) in the left temporal area that required an incremental dose of levetiracetam. No clinical seizure was observed and PLEDS eventually evolved into slowing. Five days after her admission, she regained full orientation with no noticeable neurological deficit allowing her discharge on levetiracetam 1 gram twice daily a few days later after completing 14 days of acyclovir. A repeat meningitis encephalitis panel after seven days also had negative HSV polymerase chain reaction (PCR). This was presumed to be false negative.

**Figure 3:**
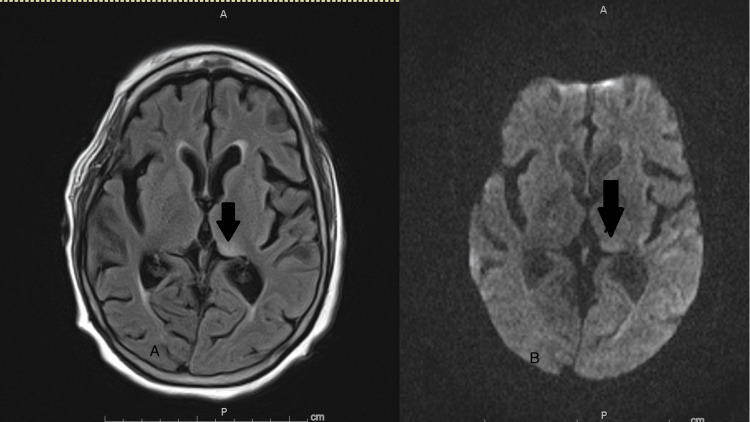
Progressive findings on MRI brain MRI brain showing area of progressing hyper-intensity affecting the posterior left thalamus in fluid attenuation inversion recovery (FLAIR) sequence (arrow in A) with a corresponding diffusion restriction (arrow in B).

**Figure 4:**
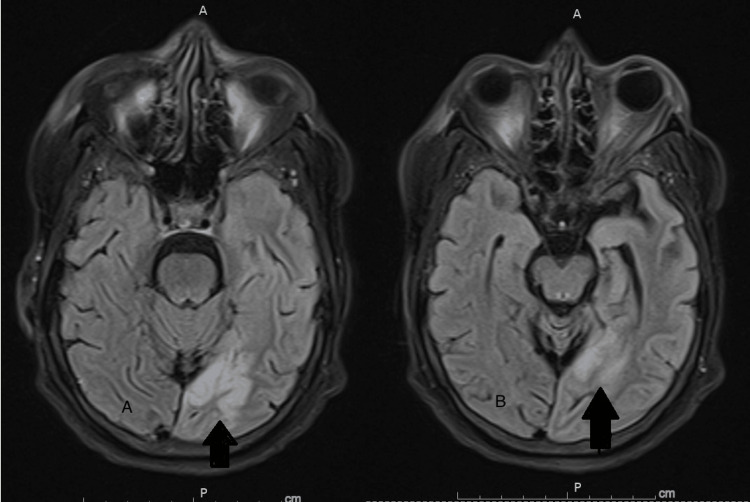
Further delineation of fluid attenuation inversion recovery (FLAIR) changes on MRI brain MRI brain showing significant hyper-intensity in FLAIR sequence affecting the left posterior parietal and occipital lobes (arrows in A and B).

**Figure 5:**
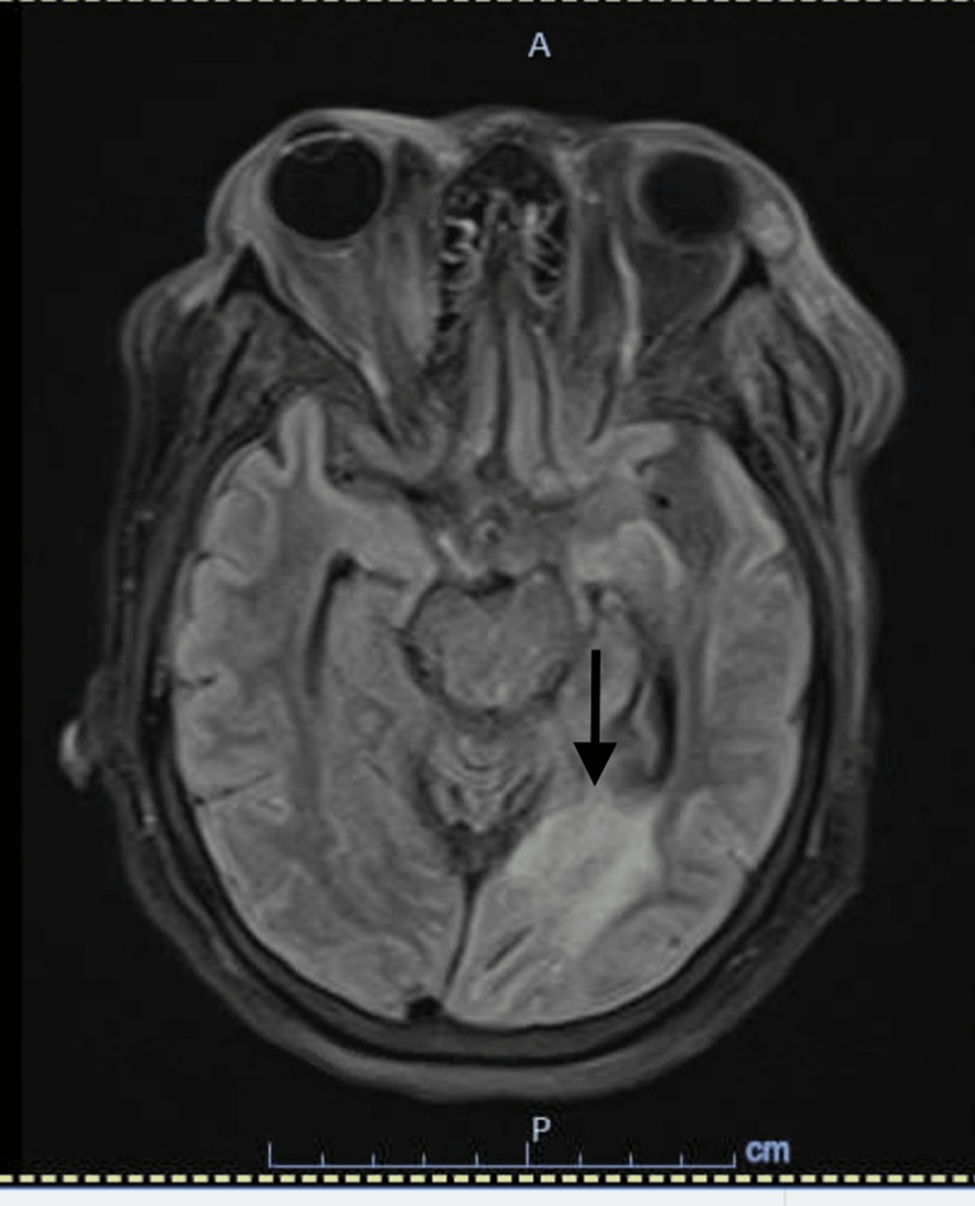
Relative improvement of fluid attenuation inversion recovery (FLAIR) changes on MRI brain MRI brain showing improvement in left posterior parietal and occipital lobes hyperintensity in FLAIR sequence (arrow).

She presented again to ED six days later with breakthrough seizure prompting increasing levetiracetam to 1.5 gram twice daily and adding clobazam. Physical exam showed mild expressive aphasia, orientation only to self, conjugate left gaze, right hemianopsia, mild right hemineglect, weakness 4/5 on the right side and hypertonia and clonus affecting right upper extremity. A repeat MRI brain FLAIR showed gyriform abnormal signal and restricted diffusion affecting the left parietal and temporal lobes involving posterior thalamus (Figures 3 and 4). 1.5-2 Hz PLEDS were seen again on EEG prompting adding lacosamide. A second LP and CSF analysis showed increasing WBC 17 cells/ml. Paraneoplastic panel including anti-Hu, anti-Yo, and anti-Ri antibodies and screening for malignancy were negative. Rheumatologic workup was negative for antinuclear antibodies (ANA), rheumatoid factor, Sjogren antibodies, and anti-dsDNA antibody. Evolution to electrographic status epilepticus (ESE) with spread to the right hemisphere despite three antiepileptic medications (AEM) prompted empiric treatment with corticosteroids and intravenous immunoglobulin (IVIG) for presumed HE as anti-thyroid peroxidase (TPO) antibody was positive. ESE persisted despite five days of IVIG prompting adding topiramate and sodium valproate. Clinically, her alertness was fluctuating with persistent neglect of the right side. PLEDS improved in frequency allowing de-escalation to 4 AEM. Extended autoimmune encephalitis panel in the CSF was positive only for anti-GAD 65 antibody titer 0.15 (reference less than 0.02). Patient remained largely obtunded with persistent right sided weakness on examination, then declined with breakthrough seizure and was started on plasmapheresis while intubated on midazolam infusion. Lack of further epileptiform discharges on EEG and interval decrease in the extent of T2 hyperintensity in the left posterior temporal and occipital lobes and restricted diffusion (Figure 5) prompted extubation. However, she remained obtunded, able to verbalize a few words, follow simple commands and localizes to noxious stimuli in all extremities. Re-emergence of ESE prompted adding phenytoin to levetiracetam, sodium valproate, and lacosamide, and starting rituximab. Neurological improvement was evident after the first dose of rituximab. She was more alert and conversant, but her speech was dysarthric. She was able to follow commands but with measurable weakness on the right side 3/5. She was also able to withdraw to noxious stimuli on four extremities. Over the next two weeks, her neurological exam continued to improve and AEM tapered down to phenytoin and levetiracetam. 



## Discussion

The incidence of autoimmune encephalitis is comparable to the incidence of infectious encephalitis, estimated at 13.7 per million per year [4]. A retrospective study found that anti-GAD encephalitis tends to present earlier than other types of autoimmune encephalitis and affects females more than males [5]. High anti-GAD titer (more than 1,000 folds of the upper normal) has higher specificity for the associated neurological disorders, although the neurological findings were highly suggestive of associated encephalitis with a low titer in our patient [2]. 

Anti-GAD antibody has been associated with seizures, cerebellar ataxia, myelopathy, brainstem dysfunction, stiff person syndrome, palatal myoclonus and encephalitis [1]. The most common is stiff person syndrome [2]. These antibodies have also been seen in association with GABA-B receptor encephalitis, which is usually associated with small-cell lung cancer and neuroendocrine tumors of the lung [6]. It is thought that they interfere with exocytosis or synthesis of GABA in the nervous system [7]. This might explain the fact that phenytoin was more effective than other AEMs in controlling patient’s seizure as phenytoin enhances the uptake of GABA. Epilepsia partialis continua has been reported in GAD encephalitis [8,9]. Initial impairment of short-term memory has also been reported [10]. The typical clinical presentation is a subacute onset (days to weeks) of cognitive decline with temporal lobe involvement [11]. Clinical presentation can range from mild confusion to dementia-like features, coma and death [12]. An interval of up to two decades between the diagnosis of temporal lobe epilepsy and diagnosis of autoimmune encephalitis has been reported [13]. 

The existence of HE has been debated recently. Its prevalence is estimated at 2.1 per 100,000 [3]. Diagnosis requires positive antithyroid antibody, good response to steroids and exclusion of other encephalopathies. There is no correlation between the titer of antithyroid antibody and the severity of HE. However, these are seen as hallmark for the diagnosis and include anti-TPO, anti-TSH receptor, antithyroglobulin and alpha enolase antibodies [3]. These are not specific and can be seen in 13% of healthy individuals and in 27% of elderly white women more than 60 years of age. There are two types of HE: vasculitic type and and indolent progressive type [14]. The first type usually presents with stroke-like symptoms and the other usually presents with psychotic features, seizure, stupor and myoclonus [14]. Seizure is seen in two-thirds of HE cases and the most common type is focal seizure with secondary generalization. New onset status epilepticus, including epilepsia partialis continua, can also be seen.

MRI is mandatory in suspected cases of autoimmune encephalitis. Usually, in anti-GAD encephalitis, the MRI shows hyperintensity in the medial temporal lobe on FLAIR and T2 sequences, which is usually asymmetrical or unilateral. However, these findings are not pathognomonic. In HE, imaging findings are nonspecific. Normal MRI does not rule out the diagnosis of both anti-GAD encephalitis and HE as this can be seen in 25% of cases [15,16]. Focal seizure, especially of medial temporal origin, is common in GAD encephalitis [2]. 

In this case, the new onset seizure, along with temporal lobe changes on imaging, led initially to confusion with herpes encephalitis as these are common features. Although CSF herpes PCR was negative twice, this did not preclude the diagnosis as false negative results have been reported [17,18]. Its sensitivity is high but it is not 100%. Causes of false negative PCR can include testing in early or late infection, low viral replication level and possible neutralization of HSV by antibodies [17]. Re-emergence of refractory seizure prompted to investigate further. However, positivity of anti-TPO antibody prompted consideration of HE. Failure to control seizure with steroids hinted toward a different diagnosis.

There is no consensus regarding treatment of anti-GAD encephalitis [19]. Therapeutic options for anti-GAD encephalitis include corticosteroids, IVIG, plasmapheresis and immunosuppressive medications including rituximab, mycophenolate mofetil and cyclophosphamide [5]. As compared to other types of autoimmune encephalitis, anti-GAD encephalitis tends to be more resistant to steroids. However, stiff person syndrome is an exception to this as it tends to be steroid-responsive [5,19]. Complete response to immunotherapy is rare. Poor prognostic findings include serum GAD-65 titer more than 500 nmol/l and cerebellar ataxia [2]. 

## Conclusions

A new onset seizure focal to temporal lobe with corresponding imaging changes can be seen in HE, anti-GAD 65 encephalitis, and herpes encephalitis. This can lead to clinical confusion. Herpes PCR has a high sensitivity and other causes of encephalitis, especially autoimmune encephalitis, should be ruled out before presuming a false negative result. HE is also a diagnosis of exclusion and failure to respond to steroids should hint toward a different etiology. Positive antithyroid antibodies can be incidental on the top of an underlying other autoimmune encephalitis.
